# Socially-Assistive Robots to Support Learning in Students on the Autism Spectrum: Investigating Educator Perspectives and a Pilot Trial of a Mobile Platform to Remove Barriers to Implementation

**DOI:** 10.3390/s22166125

**Published:** 2022-08-16

**Authors:** David Silvera-Tawil, Susan Bruck, Yi Xiao, DanaKai Bradford

**Affiliations:** 1 Australian e-Health Research Centre, CSIRO Health & Biosecurity, Brisbane 4029, Australia; 2 School of Medicine and Dentistry, Griffith University, Gold Coast 4222, Australia; 3 Autism Spectrum Australia, Frenchs Forest 2086, Australia

**Keywords:** autism spectrum disorder, social robots, socially-assistive robotics, classroom technology

## Abstract

Technology offers educators tools that can tailor learning to students’ learning styles and interests. Research into the use of socially-assistive robots as a learning support for children on the autism spectrum are showing promising results. However, to date, few schools have introduced these robots to support learning in students on the autism spectrum. This paper reports on a research project that investigated the barriers to implementing socially-assistive robot supported learning, and the expectations, perceived benefits and concerns of school teachers and therapists of students on the autism spectrum and adults on the autism spectrum. First, three focus groups were conducted with six adults on the autism spectrum, and 13 teachers and therapists of students from two autism-specific schools. During the focus groups, there was cautious optimism from participants about the value of socially-assistive robots for teaching support. While the data showed that participants were in favour of trialling socially-assistive robots in the classroom, they also raised several concerns and potential barriers to implementation, including the need for teacher training. In response to their concerns, the second part of the project focussed on developing a software platform and mobile application (app) to support the introduction of robots into autism-specific classrooms. The software platform and app were then trialled in two schools (*n* = 7 teachers and therapists). Results from focus groups indicated that participants believe socially-assistive robots could be useful for learning support, as the mobile app provides an easy to use tool to support preparing and conducting lessons that would motivate them to trial robots in the classroom.

## 1. Introduction

Technology provides teachers with novel ways to present information, and offers students opportunities to engage with topics compatible with their learning styles and interests. Consistent structured environments that provide routines, repetition and familiar behaviours and activities support learning in students with diagnosed autism spectrum disorder [1,2,3], a lifelong developmental condition characterised by difficulties with social communication and social interaction, and restricted and repetitive behaviours and interests [4].

Strengths-based learning, where the special interests and abilities of the student are incorporated into the teaching practices, can support learning, act as a motivator, and improve students’ sense of confidence in their abilities [5]. Students on the autism spectrum report they find technology less socially threatening than face-to-face social interaction, and they find playing with technology enjoyable [5,6].

Socially-assistive robots are a relatively new type of technology that employs interaction strategies, including speech, facial expressions, and body movements, to assist people in accordance with a particular context [7]. They can provide assistance by developing a social bond with individuals and supporting a measurable progression in the development of a skill [8,9,10,11]. Socially-assistive robots can interact autonomously or semi-autonomously with humans to support their daily activities [12], and have the potential to complement educators and therapists in the provision of tailored, accessible, and affordable interventions and support [13,14]. From here on, we will refer to socially-assistive robots as robots.

In education settings, robots have the potential to support learning by delivering reliable and replicable demonstrations and interactions to complement and enhance the outcomes of school lessons [13,14,15,16]. Recently, a growing body of knowledge has shown that robot involvement in therapy sessions can support social skill development in students on the autism spectrum, including verbal communication [17,18], emotion recognition [19,20], student engagement [21], and associative, cooperative, and social play [22]. Promising results were also reported on the development of social interaction abilities [19,23] and learning outcomes [24].

Small child-sized robots that have simplified human-like features and monotonous voices are generally well received by young children on the autism spectrum because they offer predictable, consistent behaviour, and a low sensory input [25]. However, the use of robots in education and clinical settings has been met with scepticism by some therapists and educators [26,27]. One reason is that the majority of research studies to date do not follow standard research designs. For example, in many studies there is a lack of a control group and sample sizes are small (often less than five participants) [28,29,30]. These small-sample studies can give valuable trends, but generalising the results (often from a single exposure to the robot) is not convincing and unlikely to present the realistic impact of robots on educational or therapeutic outcomes of young people on the autism spectrum [31].

Teachers also report concerns about their knowledge of autism and their skills in teaching students on the autism spectrum [2]. However, when accessible resources are available to support teachers and develop individualised programs, confidence and perceived knowledge improves [32]. To date, there are few professional development programs specifically designed to prepare teachers for using robots in the classroom, and there is limited information available on the perceptions and expectations of teachers of students on the autism spectrum about the introduction and use of robots in the classroom [33].

Research that explores the expectations of adults on the autism spectrum, and teachers and therapists of students on the autism spectrum, about the use of robots to assist learning and teaching is a growing area of interest for educators and policy makers. Available data suggest that educators and therapists consider this technology has the potential to be a valuable tool in the education of children and young adults on the autism spectrum [34]. However, there is very little information about the views and perceptions of individuals on the autism spectrum and a dearth of participation in the research process [35]. This study extends the existing findings and investigates the expectations, concerns, and barriers to the implementation of robots to support teaching of students on the autism spectrum, via focus groups with special education school teachers and therapists, and adults on the autism spectrum. The research questions posed in this research were:

1 What are the expectations of school teachers, therapists, and adults on the autism spectrum towards robots at school?
2 What are the concerns and barriers of school teachers, therapists, and adults on the autism spectrum towards using robots as a learning and teaching support tool?
3 What is needed to facilitate the use of robots in the classroom and improve learning opportunities?

Despite some concerns regarding the cost and fragility of robots, participants expressed considerable interest in trialling new technologies (including robots) to support learning for individuals on the autism spectrum, especially if the development of lectures was quick, simple, and did not require advanced technical skills. In response, a novel software platform and mobile application (app) were developed and trialled to enable teachers to deliver robot-assisted learning.

## 2. Methods and Materials

### 2.1. Design

This project was divided into three parts: (1) an exploratory study designed to investigate the interest from adults on the autism spectrum and educators in the use of robots to support classroom education of students on the autism spectrum; (2) the development of MAX, the first iteration of a software platform and mobile application (app) designed to facilitate the use of robots in the classroom, and (3) the pilot trial of MAX. Both the exploratory study and the pilot trial of MAX used focus groups to discuss benefits, limitations, and opportunities of robot-assisted education with the participants. A focus group discussion provides an arena for facilitator-led, qualitative data collection where the stakeholders in the research interactively discuss a particular issue to capture their opinions and perspectives [36].

The advantage of a focus group discussion is its ability to create a platform for debate about a research topic and the expression of individual participant and collective group views [37]. Focus group research is a popular method of data collection for school teacher [34,38] and school robot research [39,40]. The internal validity of the data was ensured by including the perspectives of teachers, therapists and adults on the autism spectrum [41].

This project was conducted with ethical approval from CSIRO’s Health and Medical Human Research Ethics Committee (LR 9/2017) and Aspect’s Research Approval Committee (7/2017).

### 2.2. Materials

**Figure 1:**
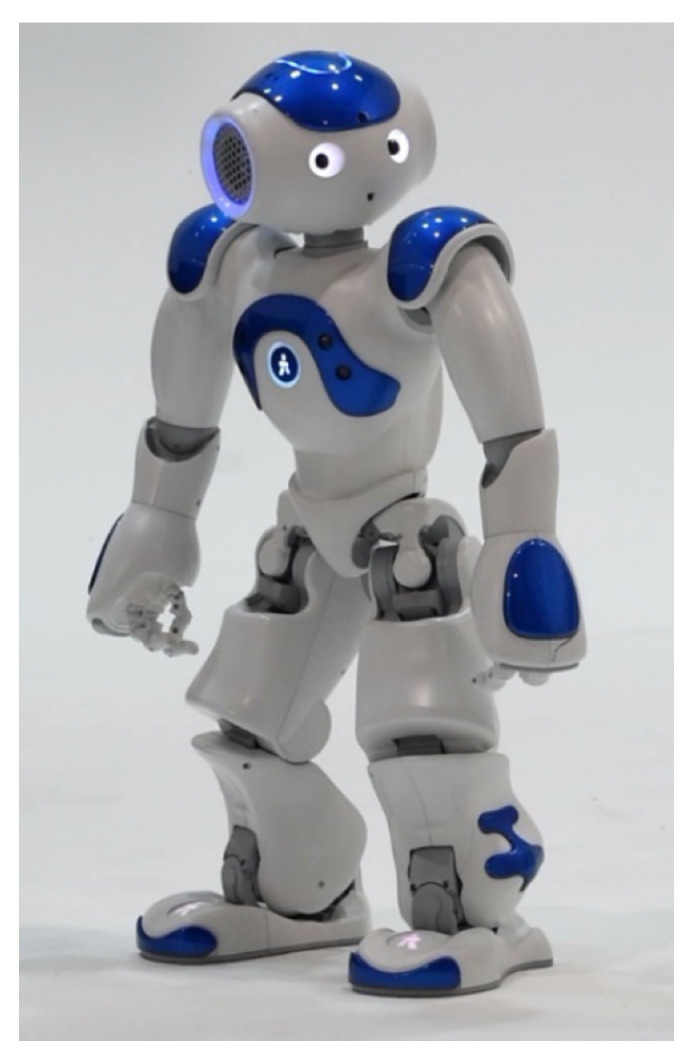
Humanoid robot NAO.

This project used NAO, a small (58 cm height, 4.3 kg), programmable humanoid robot developed by SoftBank Robotics (Figure 1). The hardware platform includes: tactile sensors, speakers, microphones, video cameras, and prehensile hands with three fingers. NAO has been used in a number of research studies with children on the autism spectrum with positive outcomes [21,42].

Informed by the exploratory study, a software platform—named MAX—was designed to facilitate teachers’ access to a robot’s functionalities, and enable the use of robots in the creation of activities to support learning. The platform includes a software library to control the NAO robot (sensors, actuators, and network operations), an Android-based mobile app, and a bi-directional communication channel between the app and the robot. While the control library was built specifically for the NAO robot, the mobile app and the communication channel were designed to work with any robot.

**Figure 2:**
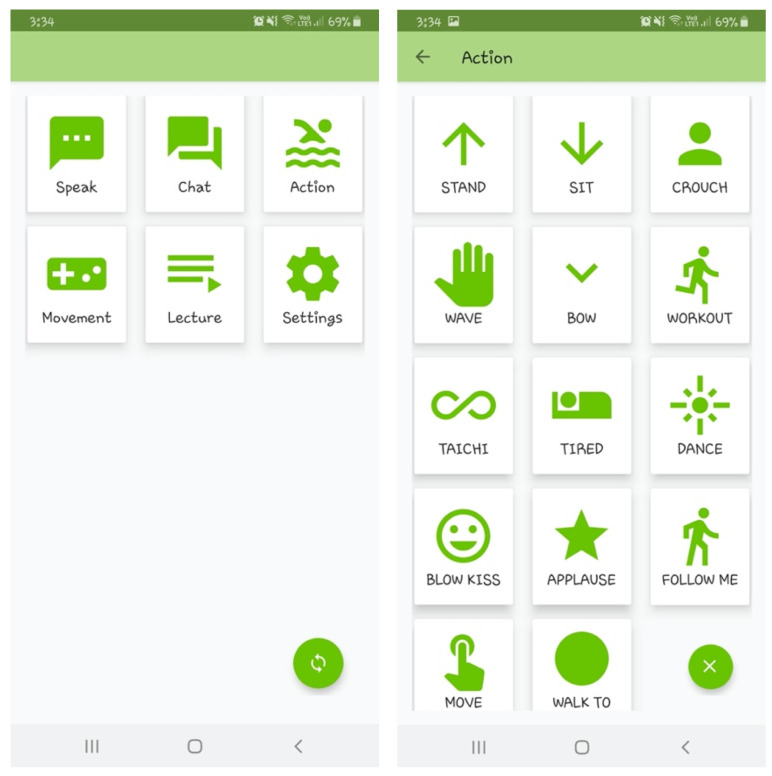
Main screen (**left**) and Action module (**right**) of the MAX app. The arrows in the bottom right corner of the main screen are used to ‘refresh’ the wireless connection, and the ’x’ in the bottom right corner of the Action module stops the current activity running on the robot.

The communication channel was made via an HTTP protocol configured to start and stop by a triple-press of the NAO’s chest button. The mobile app was developed on Android 8.0, and is compatible with Android phones and tablets. The app’s user interface (UI) design follows Google’s Material Design guidelines. The current version of the app contains a ’Settings’ section and five fully functional modules: Speak, Chat, Action, Movement, and Lecture (Figure 2). In every module, a ’Stop’ button allows the user to stop the activity running on the robot, and return full control to the user. Their features and intended use cases are briefly described in Table 1.

### 2.3. Participants

Adults on the autism spectrum, and teachers and therapists from autism-specific service providers, were invited to participate in both the exploratory study and the pilot trial of MAX. Participation was voluntary; no incentives were offered. To be included in the studies, participants needed to be at least 18 years of age. There were no exclusion criteria.

#### 2.3.1. Exploratory Study

Purposive sampling was employed in recruiting two groups of participants: (1) The Aspect Think Tank, a consultative committee whose adult members have confirmed diagnoses of autism or identify as being on the autism spectrum. (2) Aspect education staff. Aspect Think Tank members were invited to participate in the study through a post on the closed Think Tank Facebook page. Aspect teachers were invited to participate through an advertisement on the online school communication platform Yammer.

#### 2.3.2. MAX Trial

Recruitment for this study was conducted via email through a number of Australian organisations that provide education for students on the autism spectrum. To be included in the study, participants needed to be qualified therapists or educators working in the education of individuals on the autism spectrum. Invited participants were different to those who participated in the exploratory study.

### 2.4. Procedure

In all focus groups, the aims of the study and the larger project were explained prior to obtaining informed consent and informing participants of their right to withdraw. Focus groups ran for approximately 60 min each and were facilitated by one of the researchers. At least two members of the research team were present in all focus groups; one of them took hand-written notes. All focus groups were audio recorded with signed participant consent prior to commencement.

#### 2.4.1. Exploratory Study

A total of three focus groups were conducted between August and November, 2017: an initial focus group with Aspect’s Think Tank members, followed by two focus groups held with Aspect’s education staff. Focus groups started with a short (∼10 min) video demonstration of the NAO robot and a brief explanation of its capabilities. Demonstrations were followed by a semi-structured framework of questions developed to facilitate flow (Table 2), probe previous experience with robots and how a robot might assist various aspects of the curriculum (e.g., literacy and numeracy), learning processes (e.g., creative thinking), and the social environment (e.g., communication and interaction). Potential barriers and opportunities were discussed.

#### 2.4.2. MAX Trial

Two focus groups were conducted between October and November 2018: an initial focus group with therapists from non-for-profit autism-specific organisation that provides therapy and education was held in Sydney, followed by a teleconference with educators from the inclusive education centre from a public high school in regional South Australia. Both focus groups started with a short (∼5 min) demonstration of the NAO robot, followed by a short discussion of the ideal user interface to control it. Then, participants were given the opportunity to control the NAO robot using the MAX app, for approximately five minutes each. The focus group was completed with a semi-structured framework of questions aimed to collect the participants’ opinions on the current version of MAX, and its potential integration into their practice (Table 3).

### 2.5. Data Analysis

Audio recordings from both the exploratory study and the trial of MAX were transcribed verbatim and cross-checked for accuracy and missing data against the hand written notes. All data underwent a thematic analysis [45], and scripts were independently reviewed by three researchers. An inductive approach was used to determine the coding of the data, and a semantic approach to analyse the data by identifying explicit words [46]. Researchers familiarised themselves with the data, incorporated their codes, and entered their suggested themes into a spreadsheet. Then, the three researchers met to discuss the codes and to establish agreed themes.

## 3. Results

This section presents findings from the focus groups in the form of a summary. The information in this section represents the themes emerging during focus groups, as agreed by the researchers. To avoid a misleading sense of statistical validity given the non-random purposive sample and the semi-structured nature of focus groups [47,48], we refrain from reporting results using whole numbers or percentages. Instead, to provide a sense of generalizability, we describe findings as emerging from most (∼76–100% participants), many (∼51–75% participants), some (∼25–50% participants), or a few (<25% participants).

### 3.1. Exploratory Study

Six adults on the autism spectrum from the Aspect Think Tank, and 12 teachers and a school therapists from two autism specific schools—a Metropolitan School in Western Sydney and a Regional School in New South Wales, Australia—responded to the advertisement and agreed to participate (Table 4).

All participating teachers were familiar with using technology in the classroom; however, none had previous experience using robots. The focus group participants were enthusiastic, as they anticipated introducing robots as a tool to support individualised primary and high school student learning. This initial eagerness was tempered by practical concerns. Four main themes emerged from the data: (1) anticipated features that would support learning; (2) barriers to implementation, including robot cost and fragility, teachers’ limited time to attend training, and lesson preparation time; (3) general concerns, including technical challenges, sensory issues, and dependency on the robot; and (4) evaluating student outcomes. Suggestions for implementations are catalogued.

#### 3.1.1. Anticipated Support Features

Most participants were cautiously optimistic that the students would respond favourably to a robot in the classroom. They discussed the size and considered the humanoid features to likely be relatable for students.

*We could also do things such as program the robot to step back if somebody gets too close or to do some obvious body movements that we can teach the kids to read, such as, ’Look, he’s leaning back, maybe you’re too close.’* [Teacher 1]

There was consensus from most participants about the potential for student engagement, particularly when the robot was novel. The predictability and reproducibility of actions was viewed by many participants as an important feature of the robot. For students who are conscious of ‘getting it right’, a few teachers suggested that a ‘patient’ robot could be used to allow them to practice their skill in a safe setting.

Most participants also responded positively toward the suggestion of using robots with primary and high school students, and highlighted a number of features they anticipated would support learning, with the caveat that benefits may vary based on individual learning styles and needs (Table 5).

#### 3.1.2. Barriers to Implementation

Participants mentioned a number of potential barriers to implementation. These were grouped into six main categories:

Cost: Barriers around cost include the required budget for purchasing the robot, the cost of maintenance, or the time/hours of work required to prepare a lesson. The purchase price of the robot was seen as a barrier by some teachers. Other teachers were less concerned and were convinced funds were available to enable the purchase of innovative teaching tools. The cost of maintaining and repairing the robot was a recurring theme. Some participants were concerned that funding for teaching resources could be diverted to the robot program, leaving other programs underfunded.
Fragility of the robot: Many participants voiced their concerns about inconsistent WiFi connections and the reliability of the robot. The potential for damage to the robot was of significant concern:

*What happens if it gets damaged? Within the purchase price… Probably would be a warranty?’* [Think Tank member 1]

Robot availability: There was discussion about the need for predictability in the classroom and the students’ responses if the robot was unavailable due to, for example, repairs and maintenance. For some teachers, there was an expectation that there might only be one robot per school, making it a scarce resource, and perhaps not worth the effort.
Time constraints: Most participants anticipated a need to set aside significant time to develop specific programs to train students on how to work with a fragile tool. They had angst based on the need for close monitoring, supervision, and additional work; for example: how long would teachers need to prepare a lesson?
Training: Preparing lessons with the robot was perceived by most teachers to be time consuming, given that most of them do not have adequate programming skills: ’the bulk of our teachers really do not do programming at all’ [Teacher 1]. Training for all classroom educators, including teacher aides, was raised as an important issue. Teacher aides were expected to play an integral role in lesson development and maintenance of the robot.
Development of lessons: There was a suggestion (supported by most participants) that a mobile app would facilitate the preparation and delivery of lessons. In this vein, many participants agreed that a lesson hub for teachers to share programs with all teachers across different schools should exist to alleviate the load needed for software development and enable the use of the robot in classrooms.

*… once we do a program, we’ll all share it out […] and then we’d all make adjustments to it to meet our classes needs.* [Teacher 3].

#### 3.1.3. General Concerns

Participants mentioned several factors of concern directly related to the introduction of robots in the classroom:

Technical concerns: Participants mentioned several factors of concern directly related to the technology, including the battery life of the robot, how often it would need to be recharged, and how many times it could be charged before it would need replacing. These concerns were shared by most participants. Some of them also questioned the availability of solar power, and the robot’s ability to record video through its cameras. The adults on the autism spectrum raised additional concerns regarding data management and security: ’If it would be hacked, I would have concerns about the safety and security of the children who are interacting’ [Think Tank member 2]. In addition, several technical modifications were suggested (Table 6).
Robot as a reward: The use of technology as a student reward was contentious. Although many teachers suggested the robot would be a valuable reward tool that could be used to incentivize students, the Think Tank participants proffered an insight into the outcome of incentives for students on the autism spectrum. The adults on the autism spectrum explained that the robot should be considered an essential learning tool, akin to a text book. They noted that if access to the robot was only for students who met specific criteria or excelled in a particular skill, the chances were that due to their different ways of learning, some students could be disadvantaged. There was also agreement from Think Tank participants to avoid using withdrawal of the robot from the learning environment as a punishment for not achieving goals.

*… Integrating technology into lessons is rewarding because the technology itself is motivating.* [Teacher 2]

Dependency and transfer of skills: There was apprehension about the possibility of students becoming dependent on robots for learning. Some teachers stated their uneasiness about student engagement and motivation to learn, if the robot was unavailable. Concerns were raised my some participants that, for some students, the robot may become a distraction from the lesson content, especially when first introduced. Many participants were also uncertain as to whether the skills learnt with the robot were transferable to the other settings (e.g., home). Most Think Tank participants also expressed scepticism as to the value of learning social skills with a robot. They questioned whether learning to communicate with a robot is a relevant skill that translates to a practical ability needed for communicating with a human.

*My worry would be that it creates an expectation of social interaction that isn’t representative of the social interaction undertaken by your typical human.* [Think Tank member 2]

#### 3.1.4. Evaluating Student Outcomes

This theme contained participant comments around goals, individual education plans, student progress, and program evaluations that measure the impacts of robot-assisted learning. Many teachers suggested that individualised education plans (IEP) should include the robot program, generalisation of skills to the real world, and feedback from parents and/or therapists. Video content analysis was suggested by a few participants as a measure of student engagement in the robot-assisted lessons.

#### 3.1.5. Suggestions for Implementation in the Classroom

Most participants agreed that implementing a robot-assisted learning program in schools would involve meeting teacher, student, and parent expectations. These expectations would need to be managed, and all stakeholders should be made aware that the robot is implemented in the school as a tool to assist teachers in delivering content or activities, not to replace them. Robot-assisted lessons that were suggested included: social skills, life skills, navigation skills (e.g., over, under, before, behind, left, right, far, near), academic skills (e.g., mathematics and software programming), emotion regulation, and physical activity (e.g., yoga, dance). Teachers also suggested the robot may have roles in routine classroom management, role play, assessment, time keeping, and story reading.

The process of introducing the robot to students requires careful planning to ensure the students understand how to work safely with the new technology. Most participants agreed that the ‘morning circle’, or first activity of the day, was an appropriate time to introduce the robot:

*The kids can get used to it being in the morning circle, and then once they and we get comfortable with it, we can branch it out.* [Teacher 3]

There was an expectation from most participants that the more time students spend with the robot, the faster they will adapt to it as a learning tool. A number of recommendations were proposed to improve the chances of success when introducing robots to the classroom (Table 7).

### 3.2. MAX Trial

A total of three therapists (two speech pathologists and one occupational therapist) from a not-for-profit autism-specific organisation in metropolitan Sydney, and four educators from the inclusive education centre from a public high school in regional South Australia, agreed to participate (Table 8).

Participants responded positively to the potential use of an NAO in their practices. They all agreed that NAO’s non-confronting physical appearance would likely be well received by the students and teachers. They also mentioned that the three-dimensional appearance of the robot would facilitate active interactions (as opposed to passive interactions from tablets and smartphones) and possibly support skill generalisation:

*Being able to have an interaction with something [a robot] that is similar to a person […] might be closer to generalising that [with] something that is 2D [a screen].* [Therapist 1]

Some participants also suggested that a smaller version of NAO would be easier to move around different locations. The remainder of this section includes a summary of the participants’ feedback divided into: (1) the participants’ perceptions of the ideal method to deliver lectures using NAO (before the demonstration of MAX), and (2) their feedback on the current version of MAX (after the demonstration of MAX).

#### 3.2.1. A Practical Method to Deliver Lectures Using Robots

This theme relates to the participants’ perceptions of the practical method in regard to preparing and delivering lessons using the robot. Like the exploratory study, most participants suggested that a mobile app would facilitate the introduction of NAO into education. They mentioned that the app should ‘be professional; it has to look good, has to sound good … It just needs to be smooth’ [Teacher 1]. If possible, a few participants suggested that some controls should be integrated into a smart watch to reduce potential distractions from the children. Most participants also agreed that the same app should be used to control different robots, be customisable to different lectures and students, and include modules that can be executed with a single press of a button. Many participants also mentioned that the app should make it easy to prepare and personalise lectures in approximately five minutes, or up to 30 min if it involves multiple students or sessions:

*… This is going to take me half an hour to set it up, but I’m going to use it with three children then it’s worth my time… Sometimes we don’t have much time at all to plan so 5 minutes would be good.* [Therapist 2]

#### 3.2.2. Feedback on MAX

This section refers to the participants feedback on MAX, after giving them an opportunity to control NAO using the MAX app. The participants feedback was focussed around three main areas:

Usability All participants indicated that they would be willing to trial the app in the classroom, as the user interface is easy to use, the icon buttons are understandable, and the navigation is intuitive. Most of them believed the symbols and visuals were helpful, but suggest colour-coding to improve focus on the content.
Available modules All participants agreed that the available modules are a good starting point, but many of them would like to have more specific modules, such as bullying, questions and answers, turn taking, and emotion regulation. A few of them highlighted that the ‘Movement’ and ‘Actions’ modules could be useful for staff and students, but they would like to have a greater rage of actions than those currently available. While they were supportive of the ‘Speak’ and ‘Chat’ modules, some of them mentioned that the robot’s gestures, movement, and lights in the eyes might be ‘too much’ for some students, and simpler behaviours might be preferred. Finally, many participants suggested that the ‘Lecture’ module would benefit from additional methods (configurable by staff) to control the robot, including voice cues or behaviours observed through the camera.
Available features Some teachers suggested that a simpler method to connect the app to the robot was needed without the need to press the robot’s chest button: ‘… Although it [pushing the button] seems simple and straightforward, a good portion of teachers struggle with those things’ [Teacher 2]. Many participants also suggested a number of new features, including the following capabilities: (1) change the robot’s voice from the app (e.g., tone, accent, pace, etc.), (2) change the volume and pace of speech, (3) turn on and off the gestures during speech, (4) share modules through the app (i.e., a lesson hub), (5) install new robot behaviours directly from the app, (6) power on/off the robot from the app, (7) integrate sign language (e.g., Key Word Sign Australia) to provide additional cues during speech, (8) see the robot’s battery level within the app, and (9) improve the robot’s management of inflections and grammar in a sentence.

## 4. Discussion

This paper described two qualitative studies undertaken via focus groups to explore the expectations, concerns, and barriers to the implementation of robots in an education setting with students on the autism spectrum. Findings from the focus groups suggest that adults on the autism spectrum and teachers of students on the autism spectrum expect that robots could be used to assist learning and teaching. Although there were numerous concerns with the new technology, the teachers and adults on the autism spectrum were keen to discuss how robots could be integrated into the classroom to support teaching.

### 4.1. What Benefits Do Teachers Anticipate the Robot Will Offer to Teaching and Learning?

Participants’ opinions indicate that teachers foresee a wide range of opportunities for using humanoid robots in the autism-specific classroom setting. The anthropomorphic appearance and behaviour of the NAO robot meant that is was considered to be an appropriate tool for supporting the teaching of a range of skills. Additionally, the predictability of the robot and the ability to program it with discreet actions led these participants to have an expectation that robots could support student learning.

Student strengths-based learning provides positive learning settings and builds student confidence [5]. Technology has been identified as a strength in around 50% of students on the autism spectrum [5]. However, participating teachers noted that there are students who are not interested in technology or are ambivalent towards the introduction of new tools into the classroom. Hence, a slow-paced introduction of the robot to a class was considered to be a very important part of the process of implementation of robots in the classroom; different introduction schedules (from days to months) would be required for individual students depending on their developmental stages.

For some teachers, their knowledge and experience in using a wide range of technologies meant they expected that robot-assisted teaching would be an extension of the current use of technology. Teachers anticipated several learning units where they would engage the robot in the classroom—mathematics and software development were seen as natural matches. Communication and social skill development using a robot to assist students by practice were also suggested as lessons that would most likely engage students. There is previous evidence to support this notion, including the use of robots to support verbal communication [18] and emotional recognition [19]. There was also a suggestion that the robot has the potential to use alternative communication methods, such as hearing-impaired sign language (Auslan), to support students with diverse abilities. Future research should explore this possibility.

### 4.2. How Do Adults on the Autism Spectrum View Social Robots as a Learning and Teaching Support Tool?

There is limited research that has investigated the views of therapists and teachers [33], and even less research that asked people on the autism spectrum for their opinions on using technology in education [35]. A major concern for participating adults on the autism spectrum was the security and management of data collected by the robot. Specifically, the discussions covered the potential for the robot to be hacked and the security measures required to keep students safe. Student safety is essential for wide acceptance of robots in education. The adults on the autism spectrum were also keen to raise the issue of using the robot as a reward in the classroom, and were emphatic that the robot was a serious teaching tool, not a toy, and that all students should have equal access to it. By contrast, participating teachers expressed that the robot would be valuable as a reward.

There are few reports of sensory challenges with robots in students on the autism spectrum. The autistic adults in this study raised the issue of sensory sensitivities associated with the robot. They suggested that some students may find difficulties with the feel of the robot and suggested alternative covering materials as an option. The flashing LEDs of the eyes were noted as a potential source of distress and raised the option of changing the colour of the eyes as needed.

### 4.3. What Challenges and Barriers Do Participants Anticipate in Using the Robot in the Classroom?

Scepticism toward the use of robots in education and clinical settings is docume- nted [26,27]. Low uptake in schools has been limited by factors such as costs, limited range of activities, and the potential for developing a dependency on the technology [42,49,50]. Focus group participants shared these concerns. With new robot platforms coming into the market, we can expect the cost of robots to reduce significantly over the next decade, removing one of the main barriers to implementation.

Transfer of skills from robot-assisted lessons to real world settings has shown promising results [18,42]. However, participants in our focus group raised concerns about the generalisability of skills learned using robots. Teachers were worried that social interactions practised with a robot might not be representative of natural engagement and may not be transferable to other settings. There was also a concern that the classroom may be disrupted by the noise and actions of the robot, distracting students from their current tasks, thereby creating additional challenges in the classroom. The perceived fragility of the robot, the cost of repairs, and the long-term loss of the robot for repairs and maintenance, were considered major barriers to the introduction of a robot into the class.

There was apprehension about the provision of professional development and support needed to prepare lessons. In line with previous research [2,33,35], most teachers were concerned about the responsibility of creating and delivering lessons with the robot. Considering the limited professional development or training teachers receive on programming and technology, the additional time required to develop lessons and write code for the robot was seen as a significant barrier to the uptake of this technology. For those teachers who have the technical proficiency and confidence to program the robot, designing a curriculum that incorporates this technology was seen as an opportunity to develop engaging lessons. The limited time and lack of technical training were considered barriers to the introduction of robots in education.

With limited time on their hands, the introduction of robots in education appears to be challenging. Accessible resources enable teachers to have the confidence to implement inclusive teaching practices [32,51]. In response to the perceived barriers, a software platform (MAX) was developed to assist teachers with the development and delivery of lessons.

### 4.4. Facilitating Robot Technology in the Classroom

MAX is the first software platform designed to support robot-assisted education with the teacher’s needs in mind. The platform includes software to control a robot’s sensors and actuators and an Android-based mobile app that gives teachers the ability to access robot functionalities, and a number of pre-installed modules to facilitate the use of robots in the classroom. While some libraries were built specifically for the NAO robot, the mobile app and the communication channel were designed to work with any robot.

MAX reduces the time and technical skill required for preparing and delivering robot-assisted lessons, and streamlines the development of activities to support learning. The trial of the software platform and app showed that the teachers were able to control the robot, and they considered it a constructive and useful tool that would enable them to work effectively and efficiently with the robot.

Participants reported that the pre-programmed features of the MAX app such as the walk, stand and read aloud functionalities were easy to use and made the teachers reconsider the challenges of robots as teaching tools. There were several additional modules and features that participants would like to have available before the robot is introduced into their classrooms, such as customisation of speech and behaviour, integration of sign language, and inclusion of the robot’s battery level within the app. In general, the teachers stated that a fully configurable application that they could manage easily was needed, together with the ability to share new modules (or lessons) created by different teachers through a lesson hub.

There are limitations to this study. The focus groups in the exploratory study were from two schools (one metropolitan and one rural) and one Think Tank from the same autism-specific organisation. Widening the recruitment to mainstream school teachers of students on the autism spectrum may offer new insights into the expectations and technical expertise of a representative sample of teachers. Future research would also benefit from interviewing students on the autism spectrum. Furthermore, none of the participants had experience using a robot, and it is recommended that a robot be available in future explorations. The trial of MAX was limited by a small sample.

## 5. Conclusions

This paper described two qualitative studies that explored integrating robots into an education setting with students on the autism spectrum.

The article examined the perceived benefits, barriers, and concerns of adults on the autism spectrum, and teachers and therapists of students on the autism spectrum, towards the use robots in autism-specific classrooms. Despite concerns of the cost and perceived fragility of robots, there was considerable interest in involving socially-assistive robots as tools to support teaching, especially if the programming of the robot was simple. The introduction of an easy-to-use mobile app that eliminates the need for program coding skills seems to be an avenue for enabling teachers to pursue implementing robot assisted lessons. Future research in robot-assisted programs that evaluate the impact of socially-assistive robots to support development and learning in large scale longitudinal studies is necessary. 

## Figures and Tables

**Table 1:** Features of the android app and use cases.

Feature	Description	Use Case
Settings	Provides the ability to configure the robot’s name, IP address, username and password.	Initial configuration
Speak	Provides a text-to-speech interface that allows the user to input English text for the robot to speak out. While the robot speaks, random body movements are generated by the robot’s in-built animation library.	Allows users to control the robot’s conversation manually.
Chat	When the user holds the robot’s right hand, the robot’s eyes light up in green to announce that it’s ‘listening’. At the same time, it will use its microphone to perform concurrent speech recognition and generates an appropriate speech response. This module was implemented using the Google Cloud Speech API coupled with the chat-bot developed by Ireland et al. [43].	Allows users to have an interactive conversation with the robot.
Action	Facilitates access to pre-installed behaviors on the robot. The current version includes: in-built postures and animations, and open-source behaviors, such as dance sequence developed by Vernon et al. [44].	Supports lessons that involve physical activity, such as dance or yoga.
Movement	Provides a joystick-like interface to control the robot. The user can make the robot walk around and move its arms. It includes a camera view which lets the user see what the robot ‘sees’ through one of its cameras.	Conduct a teleoperated lecture, or facilitate navigation exercises.
Lecture	Allows for different blocks of text to be incorporated. When the program is started, the robot ‘reads’ out the first block of text, and then waits for a touch on its right hand before reading the next one.	Facilitates the creation of step-by-step lectures.

**Table 2:** Subset of exploratory focus group questions.

Exploratory Questions
1. Have you ever used robots in a school/education environment?
- How did you use them?
- Did you find them useful? Why?
2. We are after ideas about how robots might be used in school?
- Can you think of any specific roles?
- Would it be more effective to use it in groups, or one-to-one?
- What about involving the kids in the control of the robot?
- Would allowing them to decide how the robot looks or acts help?
3. How might a robot assist in promoting social interaction?
- Do you think students would enjoy learning with the robot?
4. How might a robot assist in promoting creative thinking?
- Do you see any benefits for promoting creativity?
5. Can you think of any issues that may arise with the use of robots?
- What about any barriers to the introduction and use of robots?

**Table 3:** Subset of MAX trial focus group questions.

Software/App Trial Questions
Pre-interaction phase with MAX
1. How would you feel about using NAO in your sessions/lectures?
2. What would be the easiest way for you to control and operate NAO?
**Post-interaction phase with MAX**
3. What do you like about the new app? Something you do not like?
4. What features or modules do you think are missing?
5. Would you feel comfortable using it in your next sessions? Why?
6. Which of the modules included would be most useful for you?

**Table 4:** Participant summary of exploratory study.

		Male	Female	Total
Aspect’s Regional School		3	5	8
Aspect’s Metropolitan School		2	3	5
Aspect Think Tank		5	1	6
	**Total**	10	9	19

**Table 5:** Perceived enabling features of the robot.

Feature	Details
Novel and engaging	Variety of programmable activities believed to be instrumental in student engagement. Anticipated higher level of engagement than with teachers.
Predictable	Messages can be delivered by robots in exactly the same way every time, reducing student’s cognitive load.
Non-judgmental	Robot is non-emotive, has no ulterior motives or preconceived expectations. Hence, students would be more likely to ‘give it a go’.
Patient	Provides opportunities to practice as often as needed. Positive comments from the robot likely to improve students’ self-esteem.
Human-like appearance and behaviour	Features and movements would be familiar and recognisable to students.

**Table 6:** Suggested technical modifications.

Recommendation	Details
Customizable appearance	Changing the robots face, mouth or eyes could provide opportunities for students to learn about emotion recognition and regulation.
Customizable covering materials	Different materials (e.g., a soft silicon-like material) may be more appealing for some students.
Customizable audio	Allow for the level of emotion and intonation in robot’s voice to be changed depending on the goals of the activity.
Alternative communication methods	Alternative communication methods would be beneficial for some students, including sign language (Auslan or Makaton), a tablet, or projections with text and images.
Artificial intelligence	The robot should: (1) identify when a student is distressed or in sensory overload; (2) understand colloquialism; (3) explain concepts in a different manner, on demand, and in a flexible way; (4) learn from the interactions between people; (5) adjust its behaviour, shut itself off, or convey information to the teacher (e.g., via a message) in ways that positively and effectively impact students.

**Table 7:** Recommendations for integrating robots into the classroom.

Recommendation	Details
Slow-paced introduction	Different students would require a different introduction schedule, from days to months, depending on their developmental stages.
Their own pace	Allow students to engage as much as they want, with the option to participate in lessons with the robot or in a traditional setting.
Manage expectations	Explain the role and availability of the robot prior to introduction into the classroom, for example, by using accessible timetables.
School-wide plan	Robots to be programmed by one teacher who runs sessions in each class, maximising efficiency and familiarity with the technology.
Control access	Staff to manage access to avoid inappropriate use. Current procedures relating to iPad use are likely to be applicable. Having access to the robot only at school would be a positive influence on learning.
From tele-operated to autonomous	A gradual transition from teacher (tele) operated interactions to computer operated (autonomous) interactions would benefit students, however, teachers should supervise all autonomous interactions and take over control if needed.

**Table 8:** Participant summary of Max trial.

		Male	Female	Total
Non-for profit		–	3	3
Inclusive education center		2	2	4
	**Total**	2	5	7

## Data Availability

Not applicable.
